# A New Approach to the Use of Kefir Grain: Potential Application as a Postbiotic in the Production of Nonfat Yogurt

**DOI:** 10.1002/fsn3.70495

**Published:** 2025-06-23

**Authors:** Fatma Çoban, Ezgi Demir Özer, Metin Yildirim

**Affiliations:** ^1^ Department of Food Engineering, Faculty of Engineering Niğde Ömer Halisdemir University Niğde Turkey; ^2^ Department of Gastronomy and Culinary Arts, School of Applied Science Cappadocia University Nevşehir Turkey

**Keywords:** dried kefir grain, gel hardness, postbiotic, viscosity, yogurt

## Abstract

The use of dried kefir grains containing kefiran with high water retention capacity in yogurt production is anticipated to improve the rheological attributes and nutritional value of yogurt and also provide a functional dimension. In addition, such an application could contribute to the utilization of surplus kefir grains leftover from traditional kefir production instead of disposing of them as waste. The aim of the current study was to investigate selected microbiological, physicochemical, and sensory characteristics of set‐type yogurt produced from reconstituted milk fortified with dried kefir grains. Four different yogurt samples were produced from milk fortified with 0% (control), 0.1%, 0.2%, and 0.3% (w/w) dried kefir grains before heat treatment. The counts of *Streptococcus thermophilus* and *Lactobacillus delbrueckii* subsp. *bulgaricus*, titratable acidity, pH, gel hardness, viscosity, water holding capacity, color (L*, a*, b*), fat, protein, and total solids content of the yogurt samples were determined after 1, 15, and 30 days of storage at 4°C. Kefir grain powder had no significant effect on the activity of *S. thermophilus* (8.50–9.84 log cfu/g) and *L. delbrueckii* subsp. *bulgaricus* (8.48–9.39 log cfu/g), pH (4.17–4.43), titratable acidity (1.31%–1.41%), viscosity (1995–2302 mPa·s), water holding capacity (94.91%–96.42%), or sensory qualities of the experimental yogurts but significantly reduced the hardness values (340.5–456.6 g). The addition of kefir grain powder significantly decreased the a* values of the yogurt samples, resulting in a greener color. Unexpectedly, the viability of yogurt bacteria was not altered by the addition of kefir grain powder. However, the yogurts produced with the addition of kefir grain powder were assigned slightly higher scores by the panelists. Our results support the hypothesis that kefir grain powder may be used as a potential postbiotic ingredient in producing yogurt that is more appealing to health‐conscious consumers.

## Graphical abstract

Microbiological, physicochemical, and sensory properties of set type yogurts produced from milk fortified with different proportions of dried kefir grains were compared during a 30‐day storage period. The results supported the hypothesis that kefir grain powder may be used as a potential postbiotic ingredient in producing yogurt that is more appealing to health‐conscious consumers, without significantly altering the physicochemical properties of yogurt.

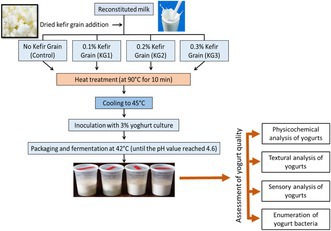

## 1 Introduction

Yogurt is a fermented dairy product with high nutritional value that is produced as a result of lactic acid fermentation by *Lactobacillus delbrueckii* subsp. *bulgaricus* and *Streptococcus thermophilus* . It is a good source of protein, fat, vitamins, calcium, and phosphorus. Human studies have shown that yogurt consumption increases antibody formation, cytokine synthesis and various cell activities, all of which help fight diseases (Azam et al. 2023). Rheological properties such as serum separation, gel hardness, and viscosity are the main factors altering the quality of fermented dairy products, including yogurt (Tamime and Robinson 1999; Özer 2006).

Like yogurt, kefir is a fermented dairy product that contains a wide variety of bioactive compounds such as organic acids, peptides, and exopolysaccharides (de Oliveira Leite et al. 2013). Kefir may improve the immune system by increasing the production of macrophages, B cells, T cells and neutrophils (Azam et al. 2023). The natural and traditional starter culture used in kefir production involves kefir grains that are separated by filtration following fermentation. Kefir grains are an interactive ecosystem of microorganisms, including lactic acid bacteria, yeasts, and acetic acid bacteria, that are immobilized on a carbohydrate and protein matrix (Farnworth and Mainville 2008; Nejati et al. 2020). Kefir grains, ranging in diameter from 0.3 to 3.5 cm (Marshall 1993; de Oliveira Leite et al. 2013), mainly consist of exopolysaccharide kefiran, proteins and microbial cell and cell debris (Nejati et al. 2020). Schoevers and Britz (2003) reported that kefir grains contain 82.6%–83.5% moisture, 4.61%–5.43% protein, 1.35%–1.69% fat, and 6.34%–7.76% total carbohydrates. Grains grow, multiply and in the meantime, constantly produce new generations of grains (Simova et al. 2002). This causes the formation of surplus kefir grains, which are considered waste.

Kefiran is a branched, water‐soluble exopolysaccharide containing equal amounts of glucose and galactose (Kooiman 1968; Gentry et al. 2023). It has attracted attention as a potential food additive because of its high water holding capacity, antifungal (Cevikbas et al. 1994), antibacterial, and antioxidant activities; and epithelium protection and immunomodulation properties (Maeda et al. 2004; Güzel‐Seydim et al. 2005; Piermaria et al. 2008; Moradi and Kalanpour 2019).

The expert panel of the International Scientific Association for Probiotics and Prebiotics (ISAPP) defined postbiotics as “preparations of inanimate microorganisms and/or their components that confer a health benefit on the host” (Salminen et al. 2021). According to this definition, kefir grains inactivated and dried by various methods can be considered a valuable postbiotic, because kefir grains, which are mainly composed of the exopolysaccharide kefiran, proteins and various microbial cells and cell residues (Nejati et al. 2020), can provide health benefits to the host like kefir. Fortifying traditional foods such as yogurt with postbiotic powders is an effective approach for the production of functional food products. However, preserving both the functional properties of postbiotic powders and the physical properties of the fortified products during this process is essential (Yousefvand et al. 2024). Recently, postbiotics obtained from *Lactobacillus acidophilus* LA5 and *Bifidobacterium animalis* subsp. *lactis* BB‐12 have been used in the production of functional foods, especially in dairy products (Sharafi et al. 2022; Sadighbathi et al. 2023; Yousefvand et al. 2024). The use of dried kefir grains containing proteins, various microbial cells and cell residues and kefiran with high water retention capacity in yogurt production is anticipated to improve the rheological attributes and nutritional value of yogurt and also provide a functional dimension. In addition, such an application could contribute to the utilization of surplus kefir grains leftover from traditional kefir production instead of disposing of them as waste. To the best of the researchers' knowledge, there are no studies in the literature on the use of dried kefir grains in yogurt production. Therefore, the main purpose of this study was to examine the effect of lyophilized kefir grains on some selected microbiological, physicochemical and sensory attributes of yogurt.

## 2 Materials and Methods

### 2.1 Microorganisms and Supplements

Traditional kefir grains were donated by the Ankara University Faculty of Agriculture Milk Processing Unit (Ankara, Turkey). Lactobacilli, lactococci and yeast contents of kefir grains were 9.05, 8.87, and 6.55 log cfu/g (Güzel‐Seydim et al. 2005). The skim milk powder used in yogurt production was obtained from Bakkalbasioglu Dairy Plant (Niğde, Turkey). Yogurt culture (Y 412) containing *S. thermophilus* and *L. delbrueckii* subsp. *bulgaricus* was purchased from Maysa Gida (Istanbul, Turkey) (starter culture was used after being activated twice in reconstituted milk with 10% w/v, total milk solids). The ultra‐high temperature (UHT) skim milk (0.1% w/w, fat) used in the production of kefir grains was purchased from the retail market in Niğde (Turkey). The screw‐capped polypropylene containers (100 mL) used in the packaging of yogurt were procured from Firat Plastic (Istanbul, Turkey).

### 2.2 Preparation of Kefir Grains as Postbiotic

The optimum inoculation rate during the propagation process of kefir grains was determined through preliminary experiments. For this purpose, 4%, 6%, and 10% (w/v) inoculation levels were examined at 24°C for 24 h, and the daily weight gain rates of the kefir grains were approximately 1.52 g/100 mL, 1.80 g/100 mL, and 2.20 g/100 mL, respectively. Consequently, the 10% inoculation rate, which resulted in the highest yield, was used to propagate kefir grains for the later stages of the research. The kefir grains were propagated by adding 10% (w/v) kefir grains to UHT skim milk, followed by incubation at 24°C for 24 h with shaking at 90 rpm (St 30 shaking water bath, Nuve, Ankara, Turkey). After incubation, the resulting kefir grains were collected by filtration through a strainer with a pore diameter of approximately 1 mm, washed twice with pure water, and eventually frozen at −86°C. Frozen kefir grains were lyophilized (ScanVac CoolSafe Pro95‐15; LaboGene ApS, Lynge, Denmark), ground with a coffee grinder (Premier Prg 259‐170; Nasmina Inc., Istanbul, Turkey), sieved (< 0.15 mm) and subsequently stored at −80°C until use. Before the use of dried kefir grain in yogurt production, a 6% (w/w) dispersion was prepared with distilled water in an airtight glass jar and heated at 75°C for 10 min. The mixture was then cooled to room temperature and used within 2 h.

### 2.3 Production of Yogurt With Postbiotics

**FIGURE 1:**
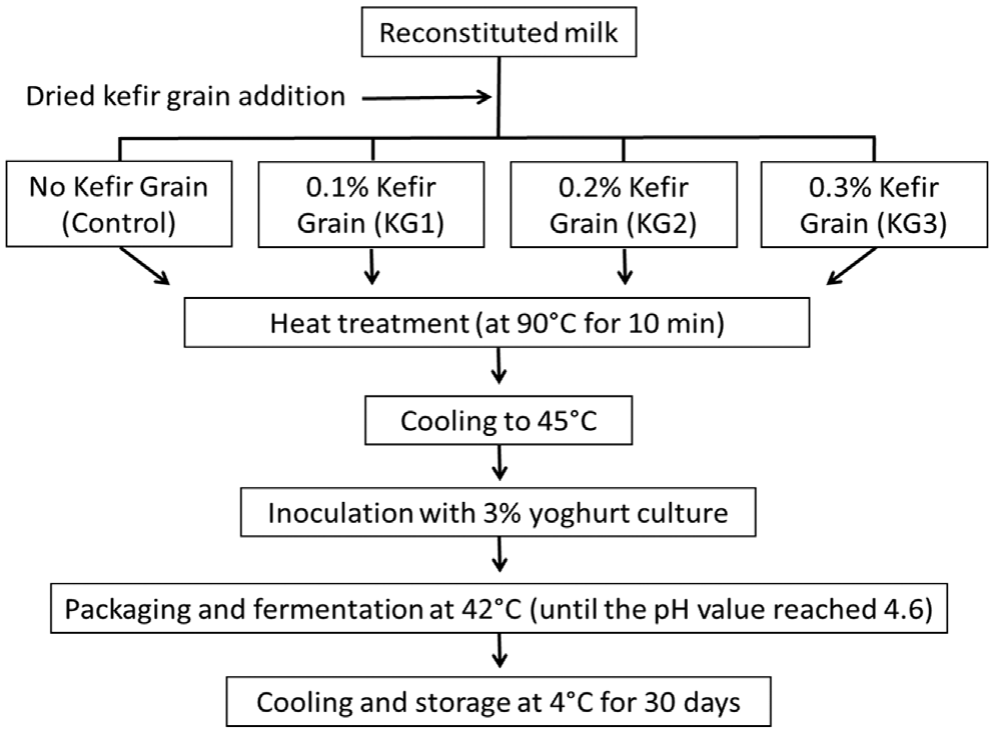
Yogurt manufacturing flowchart. Refer to Table 1 for yogurt formulations.

In nonfat yogurt (< 0.5% milk fat) production, reconstituted milk with 15.5% total solids, prepared by dispersing skim milk powder in distilled water, was used. Once the dispersion process was complete, the reconstituted milk was kept at refrigerator temperature for approximately 24 h to ensure complete hydration of the milk solids and was filtered through a double‐layered cheesecloth with a pore diameter of approximately 1 mm just before use. In the study, four groups of yogurt containing 15% (w/w) total solids and different proportions of dried kefir grains (control: 0%, KG1: 0.1%, KG2: 0.2%, and KG3: 0.3%, w/w) were produced via the formulations given in Table 1 and the process outlined in Figure 1. These dried kefir grain concentrations were chosen on the basis of preliminary experiments in which concentration ranging from 0.1% to 1.5% were tested. The yogurt milk base formulations were heat treated in a water bath at 90°C for 10 min. After heat treatment, the bases were rapidly cooled to approximately 45°C, after which 3% yogurt culture was added, the samples were subsequently transferred to 100 mL polypropylene containers and eventually incubated at 42°C in an incubator (IB 15G; Jeio Tech. Ltd., Seoul, South Korea) until the final pH reached 4.6. During incubation, pH was monitored by keeping a pH electrode immersed in a yogurt container from each of the four groups. Once the target pH value was reached, the yogurt samples were transferred to a refrigerator at 4°C. The yogurt samples were subjected to physicochemical and microbial analyses on Days 1 (24 h after production), 15, and 30 of storage and to sensory analyses only on Day 15. The production of yogurts and their analyses were repeated three times within 6 months.

**TABLE 1:** The composition of yogurt milk bases.

Component	Control (0.0%)	KG1 (0.1%)	KG2 (0.2%)	KG3 (0.3%)
Dried kefir grain solution (6%, w/w) (g)^a^	0	1.7 (0.1)	3.3 (0.2)	5.0 (0.3)
Reconstituted milk (15.5%, w/w) (g)^b^	96.8 (15.0)	96.1 (14.9)	95.5 (14.8)	94.8 (14.7)
Distilled water (g)	3.2	2.2	1.2	0.2
Total mass (g)^c^	100.0 (15.0)	100.0 (15.0)	100.0 (15.0)	100.0 (15.0)

^a^ Total kefir grain solids (g) are given in parentheses.

^b^ Total milk solids (g) are given in parentheses.

^c^ Total solids (g) are given in parentheses.

### 2.4 Analyses of Skim Milk Powder and Dried Kefir Grain

Total solids, protein, fat, and carbohydrate analyses of skim milk powder and kefir grain powder were performed via near‐infrared reflectance spectroscopy (Perten DA‐7200; Huddinge, Sweden). The samples were directly scanned after being spread into the metal containers of the near‐infrared reflectance spectroscopy.

### 2.5 Analyses of Yogurts

#### 2.5.1 Physicochemical Analysis

The pH values of the yogurts were measured using a digital pH meter (pHenomenal 1000L; VWR International Ltd., Dublin, Ireland). The titratable acidity (as %lactic acid), total solids and fat content of yogurt samples were determined according to the methods specified in Turkish Standard 1330 (TSI 2006). The protein content of the samples was analyzed via the Kjeldahl method (IDF 1993).

#### 2.5.2 Color

The CIELAB parameters (L*, a*, b*) of the yogurt samples were determined from three different locations under C light using a CR‐400 Minolta colorimeter (Konica Minolta Optics Inc., Tokyo, Japan). In this system, the a* value ranges from green (−) to red (+), the b* value ranges from blue (−) to yellow (+), and the L* value is an estimation of lightness varying from 0 (black) to 100 (white).

#### 2.5.3 Gel Hardness

The gel hardness of the yogurt samples was determined by the YOG1/BEC method recommended by Stable Micro Systems Ltd. using a texture analyzer (TA‐XTplus; Stable Micro Systems Ltd., Godalming, England) (with 5 kg load). Briefly, the measurement was carried out at 10°C on the sample in a plastic container with a diameter of 5.5 cm and a depth of 6.8 cm by inserting the probe (diameter: 35 mm and thickness: 5 mm) to a depth of 30 mm at a speed of 1 mm/s. After the test, the maximum force in the force‐time graph was used as the measure of hardness (Bozan 2023).

#### 2.5.4 Gel Viscosity

The viscosity of the yogurt samples was determined via a viscometer (DV2Textra; Brookfield AMETEK, Middleborough, MA, USA). First, the yogurt samples in the plastic container (5.5 cm in diameter and 6.8 cm in depth) were mixed with a hand mixer for 1 min and recovered for 5 min in the container, and then their viscosities at 10°C were measured using the number 64 probe (with LVK protector) at 40 rpm for 3 min. The results obtained in the first minute of the measurement were considered the viscosity (mPa·s) of the samples (Bozan 2023).

#### 2.5.5 Water Holding Capacity (WHC)

For the water holding capacity analysis, the method of Gauche et al. (2009) was used with minor modifications. After mixing with a hand mixer for 1 min, 30 g of yogurt sample was placed in a test tube and then centrifuged (NF 800R; Nuve, Ankara, Turkey) at 350 × *g* for 10 min at a temperature of 10°C. The supernatant was subsequently discarded, and the residue was weighed. The WHC values (%) of the yogurt samples were calculated with the following formula:

$$\text{Water holding capacity}\;\left(\%\right)=\frac{\text{Weight of residue}\;\left(g\right)\times 100}{\text{Initial weight of yogurt}\;\left(g\right)}$$

### 2.6 Microbiological Analysis

#### 2.6.1 Enumeration of *Streptococcus thermophilus*

M‐17 agar was used for the enumeration of *S. thermophilus* . Ten grams of each yogurt sample was mixed with 90 mL of peptone solution (0.1%) using a vortex mixer, and 1/10 dilutions were prepared with the same peptone solution. After that, 0.1 mL of the dilutions was spread on M‐17 agar and incubated at 37°C for 48 h in an incubator (IB 15G incubator; Jeio Tech. Ltd., Seoul, South Korea). Finally, the colonies formed were counted on plates containing 30–300 colonies and expressed as log colony forming units per gram of yogurt (log cfu/g) (TSI‐ISO 2004).

#### 2.6.2 Enumeration of *Lactobacillus delbrueckii* subsp. *bulgaricus*

De Man, Rogosa, Sharpe (MRS) agar was used for the enumeration of *L. delbrueckii* subsp. *bulgaricus*. Ten grams of each yogurt sample was mixed with 90 mL of peptone solution (0.1%) via a vortex mixer, and 1/10 dilutions were prepared with the same peptone solution. After that, 0.1 mL of the dilutions were spread on MRS agar and incubated anaerobically (CCL‐170B‐8 CO_2_ incubator; Esco Micro Pte Ltd., Singapore) at 37°C for 72 h. Finally, the colonies formed were counted on plates containing 30–300 colonies and expressed as log colony forming units per gram of yogurt (log cfu/g) (TSI‐ISO 2004).

### 2.7 Sensory Analysis

Sensory evaluation was carried out with a group of nine semi‐trained panelists consisting of five female and four male members between the ages of 20 and 50. Panelists were selected based on age, gender and familiarity with yogurt. To ensure consistency, panelists received brief training on the evaluation protocols. A multi‐sample difference test (rating approach) was used in the evaluation (Meilgaard et al. 1999). Yogurt samples were served in 100 mL transparent plastic containers labeled with 3‐digit random numbers. In each session, four samples were evaluated simultaneously. Evaluators tested the samples on the 15th day of storage using a 9‐point hedonic scale ranging from 1 (dislike at all) to 9 (like very much).

### 2.8 Statistical Analysis

The study was conducted in a randomized block design with three replications. Statistical evaluation and comparison of the data obtained for yogurt's physicochemical, microbiological and sensory evaluations were performed using one‐way ANOVA (*p* ≤ 0.05) in the SPSS program (SPSS Windows, Version 15.0; SPSS Inc., Chicago, IL, United States). The difference between the treatment means was determined via Duncan's multiple range test (*p* ≤ 0.05).

## 3 Results and Discussion

### 3.1 Composition of Skim Milk Powder and Dried Kefir Grain

The chemical composition of the skim milk powder used in yogurt production was as follows: 94.52 ± 0.25% total solids, 35.94 ± 0.51% protein, 1.99 ± 0.03% fat, 48.13 ± 0.13% carbohydrate (lactose), and 7.18 ± 0.24% ash. However, the chemical composition of the kefir grain powder was as follows: 91.50 ± 0.11% total solids, 31.65 ± 1.17% protein, 2.57 ± 0.55% fat, 48.41 ± 0.48% carbohydrate, and 10.57 ± 0.44% ash.

### 3.2 Physicochemical Attributes of Yogurt Samples

#### 3.2.1 pH Values

The main function of lactic acid fermentation in yogurt production is to convert milk into yogurt gel by lowering the pH to 4.6. The pH value determines the sensory qualities of yogurt and affects its shelf‐life. The pH values of the yogurt samples assessed on the 1st, 15th, and 30th days of storage at 4°C are given in Table 2. As shown in the table, the pH values of the samples varied between 4.17 and 4.43, with the control yogurt having the lowest value. Statistical analysis revealed that the effect of storage time on pH was significant (*p* ≤ 0.05), but the effect of the addition of dried kefir grain was not significant (*p* > 0.05). During storage, no relationship was observed between the changes in pH values of the trial yogurts and the levels of added kefir grain powder, which was inconsistent with the findings of Sadighbathi et al. (2023), Yousefvand et al. (2024) and Pham et al. (2024), who reported significant decreases in pH values in samples containing postbiotic powders compared with control samples. It is assumed that this difference is due to the fact that these researchers used much more postbiotic powders in yogurt production compared to our study. Therefore, it was concluded that the addition of kefir grain powder as a postbiotic neither stimulated nor retarded the growth of yogurt bacteria. On the other hand, the pH values of the yogurt samples decreased gradually but significantly (*p* ≤ 0.05), mainly because lactic acid was produced by starter culture as the storage period progressed, a trend consistent with the findings of previous studies (Doleyres et al. 2005; Sadighbathi et al. 2023; Pham et al. 2024; Yousefvand et al. 2024).

**TABLE 2:** Changes in pH, titratable acidity (as % lactic acid), total solids (%), fat (%), and protein content (%) of yogurt samples during storage.

Analyses	Yogurt types	Day of storage		
		1	15	30
pH	C^1^	4.43 ± 0.02	4.34 ± 0.01	4.17 ± 0.01
	KG1	4.41 ± 0.09	4.35 ± 0.00	4.19 ± 0.01
	KG2	4.43 ± 0.09	4.35 ± 0.04	4.22 ± 0.01
	KG3	4.43 ± 0.10	4.35 ± 0.02	4.25 ± 0.00
	Mean^2^	4.43 ± 0.03^a^	4.35 ± 0.03^b^	4.21 ± 0.03^c^
Titratable acidity (as %lactic acid)	C^1^	1.31 ± 0.02	1.35 ± 0.01	1.41 ± 0.02
	KG1	1.35 ± 0.00	1.34 ± 0.02	1.39 ± 0.03
	KG2	1.34 ± 0.00	1.32 ± 0.04	1.35 ± 0.07
	KG3	1.33 ± 0.02	1.34 ± 0.01	1.37 ± 0.01
	Mean^2^	1.33 ± 0.01^a^	1.34 ± 0.01^a^	1.38 ± 0.01^b^
Total solids (%)	C^1^	15.00 ± 0.10	15.19 ± 0.39	15.25 ± 0.29
	KG1	14.80 ± 0.26	15.09 ± 0.25	15.22 ± 0.29
	KG2	14.75 ± 0.34	15.06 ± 0.17	15.21 ± 0.26
	KG3	14.69 ± 0.45	15.11 ± 0.23	15.18 ± 0.29
	Mean^2^	14.81 ± 0.13	15.11 ± 0.13	15.22 ± 0.13
Fat (%)	C^1^	0.00 ± 0.00	0.05 ± 0.02	0.08 ± 0.04
	KG1	0.10 ± 0.00	0.04 ± 0.03	0.08 ± 0.04
	KG2	0.05 ± 0.07	0.05 ± 0.04	0.05 ± 0.07
	KG3	0.03 ± 0.04	0.06 ± 0.04	0.08 ± 0.04
	Mean	0.05 ± 0.02	0.05 ± 0.02	0.07 ± 0.05
Protein (%)	C^1^	4.84 ± 0.10	4.94 ± 0.00	4.99 ± 0.10
	KG1	5.12 ± 0.31	4.94 ± 0.05	5.02 ± 0.11
	KG2	5.05 ± 0.19	4.81 ± 0.17	5.03 ± 0.11
	KG3	4.69 ± 0.30	4.95 ± 0.04	5.06 ± 0.11
	Mean^2^	4.93 ± 0.08	4.91 ± 0.08	5.03 ± 0.08

*Note:* Values with different letters (a–c) in the same row are significantly different (*p* ≤ 0.05). C: control, KG1: 0.1%, KG2: 0.2%, and KG3: 0.3% kefir grain containing yogurt sample.

^1^ Mean ± standard deviation.

^2^ Mean ± standard error.

#### 3.2.2 Titratable Acidity (as % Lactic Acid)

The titratable acidity levels of yogurts are altered by factors such as starter culture strains used in production, incubation and storage conditions, and total solids and protein contents. The changes in the titratable acidity (% lactic acid) of yogurt samples on the 1st, 15th, and 30th days of storage are specified in Table 2. The titratable acidity of the yogurts varied between 1.31% and 1.41%, with the control yogurt having the highest value. The addition of kefir grain powder did not have a significant effect on the titratable acidity values during storage (*p* > 0.05), which was interpreted as the addition of kefir grain powder having no effect on the growth of yogurt starter bacteria or the buffering capacity of yogurt. Titratable acidity increased significantly (*p* ≤ 0.05) in all yogurt types as the storage time progressed, coupled with a simultaneous decrease in pH, indicating gradual lactic acid production by starter bacteria (post‐acidification). The titratable acidity values of experimental yogurts in the present study were similar to the values reported by Sadighbathi et al. (2023) and Yousefvand et al. (2024) because post‐acidification is a common observation in yogurts.

#### 3.2.3 Total Solids Content

The total solids content of yogurt is crucial in terms of quality, as it affects the physical and sensory properties of yogurt gels. The changes in the total solids content of the yogurt samples during storage are presented in Table 2. The total solids content was adjusted to 15% in all trial yogurt samples to eliminate its effects on the physical and chemical attributes of yogurt gel. Throughout storage, minor fluctuations in total solids content were detected in all yogurt formulations. As shown in the table, the total solids content of the samples varied between 14.69% and 15.25%, with the KG3 sample having the lowest value. Statistical analysis indicated that the addition of kefir grain powder had an insignificant effect on the total solids content (*p* > 0.05). Moreover, the storage duration had an insignificant effect on the total solids content (*p* > 0.05). This insignificant change in the total solids content during storage was quite expected since the samples were packaged in a way to prevent water loss, and no treatment was applied that would alter the total solids content. Our findings for total solids were similar to those reported by Sadighbathi et al. (2023), but much higher than those reported by Yousefvand et al. (2024) and Pham et al. (2024) for yogurts with added postbiotic powders.

#### 3.2.4 Fat Content

All the yogurts in this study had very low fat contents because they were produced from a milk base prepared with skim milk powder. As can be seen from Table 2, the fat content of the experimental yogurts varied between 0.0% and 0.10% during storage. Statistical analysis revealed that neither the addition of kefir grain powder nor the storage period had a significant effect on the fat content of the trial yogurts (*p* > 0.05). This result was quite expected since the samples were packaged in a way to prevent water loss, and no treatment was applied that would influence the fat content.

#### 3.2.5 Protein Content

Protein content is important in terms of affecting both the textural and sensory qualities of yogurt. The protein contents of the trial yogurts varied between 4.69% and 5.12% throughout storage, with the highest value being determined in the KG1 sample (Table 2). Statistical analysis revealed that neither the kefir grain powder addition nor the storage time markedly altered the protein content of the trial yogurts (*p* > 0.05). This result was highly anticipated for the following reasons. The incorporation of kefir grain powder caused an insignificant change in the protein content of the trial yogurts, as it replaced skim milk powder containing approximately the same amount of protein. Furthermore, there was no treatment that would cause a change in the protein content of the yogurt samples during the entire storage period. The protein contents of all yogurt formulations during storage were similar to the values of 4.10%–5.05% reported by Isleten and Karagul‐Yuceer (2006) and 4.62%–5.00% reported by Delikanli and Ozcan (2017) and higher than the values of 3.76%–3.87% reported by Akın and Akın (2016).

### 3.3 Changes in Color Parameters

The lightness/darkness (L*) scores of the experimental yogurt samples determined according to the CIELAB color system are presented in Table 3. The L* values of the trial yogurts varied between 90.83 and 93.44 throughout storage, with the KG2 sample having the lowest value. The L* values of the yogurt samples fluctuated irregularly throughout storage. In line with this observation, statistical analysis revealed that neither the addition of dried kefir grain nor the storage duration had a substantial effect on the L* values (*p* > 0.05).

**TABLE 3:** Changes in color parameters of yogurt samples during storage.

Criteria	Yogurt types	Day of storage			Mean^2^
		1	15	30	
L*	C^1^	91.62 ± 0.49	91.19 ± 0.16	92.21 ± 1.39	91.67 ± 0.50
	KG1	91.50 ± 0.42	91.66 ± 0.86	91.81 ± 0.27	91.66 ± 0.50
	KG2	92.37 ± 0.65	90.83 ± 0.38	91.58 ± 0.96	91.59 ± 0.50
	KG3	93.44 ± 1.40	91.74 ± 0.75	91.40 ± 1.29	92.19 ± 0.50
	Mean^2^	92.23 ± 0.43	91.35 ± 0.43	91.75 ± 0.43	
a*	C^1^	−4.32 ± 0.22	−4.72 ± 0.01	−3.75 ± 0.27	−4.26 ± 0.17^A^
	KG1	−4.73 ± 0.17	−4.91 ± 0.15	−4.16 ± 0.48	−4.60 ± 0.17^AB^
	KG2	−4.82 ± 0.25	−4.98 ± 0.09	−4.46 ± 0.57	−4.76 ± 0.17^B^
	KG3	−4.71 ± 0.17	−4.21 ± 0.37	−4.03 ± 0.29	−4.32 ± 0.17^A^
	Mean^2^	−4.64 ± 0.14^a^	−4.70 ± 0.14^a^	−4.10 ± 0.14^b^	
b*	C^1^	10.56 ± 0.36	10.08 ± 0.45	9.56 ± 0.98	10.07 ± 0.48
	KG1	11.24 ± 0.71	12.26 ± 0.01	9.38 ± 0.87	10.96 ± 0.48
	KG2	11.35 ± 1.05	10.33 ± 0.55	9.68 ± 1.14	10.45 ± 0.48
	KG3	10.90 ± 0.77	9.87 ± 0.34	9.59 ± 1.56	10.12 ± 0.48
	Mean^2^	11.01 ± 0.42^a^	10.63 ± 0.42^a^	9.55 ± 0.42^b^	

*Note:* Values with different letters (A, B) in the same column are significantly different (*p* ≤ 0.05). Values with different letters (a, b) in the same row are significantly different (*p* ≤ 0.05). C: control, KG1: 0.1%, KG2: 0.2%, and KG3: 0.3% kefir grain containing yogurt sample.

^1^ Mean ± standard deviation.

^2^ Mean ± standard error.

The a* coordinate values of all the yogurt samples were negative (green tonalities) and varied between −4.98 and −3.75 during storage (Table 3). Although the a* values of the yogurt samples increased or decreased irregularly during storage, statistical analysis showed that the effect of storage time and dried kefir grain addition on the a* values was highly significant (*p* ≤ 0.001). The data in Table 3 show that the KG2 yogurt sample containing 0.2% kefir grain powder was similar to the KG1 yogurt sample in terms of green color (*p* > 0.05) but was greener than the control and KG3 yogurt samples (*p* ≤ 0.05).

The b* values of the yogurt samples throughout storage varied between 9.38 and 12.26 (Table 3), with the KG1 sample having the lowest value. Throughout storage, the b* values of all the trial yogurt samples (except for the KG1 sample) continuously decreased. In other words, as storage progressed, the yogurt samples became less yellow. The statistical analysis confirmed that the effect of storage time on b* values was significant (*p* ≤ 0.05), whereas the effect of kefir grain powder addition was insignificant (*p* > 0.05). Accordingly, it was determined that the yogurt samples had significantly lower b* values on the 30th day of storage than those in the other two periods (1st and 15th days), that is, they were significantly less yellow (*p* ≤ 0.05).

### 3.4 Gel Hardness

One of the important factors affecting consumer preference for set‐style yogurt is the hardness of the gel. The gel hardness of all the tested yogurt samples ranged from 340.5 to 456.6 g throughout the cold storage (Table 4). The hardness value was notably affected by both the addition of dried kefir grain and the storage period (*p* < 0.001). The control yogurt had a significantly greater hardness value (425.4 g) than the other experimental yogurt did (*p* ≤ 0.05). As the amount of kefir grain powder increased, the hardness values of the experimental yogurts decreased significantly (*p* ≤ 0.05). The most likely reason for the significant decrease in the hardness of the test yogurts with increasing kefir grain powder addition was that the polysaccharide (kefiran) component of the kefir grain powder penetrated between the casein molecules and weakened the gel matrix. Hydrodynamic studies have shown that caseinates and kefiran molecules are incompatible and do not interact with each other (Exarhopoulos et al. 2023). A similar result was reported by Doleyres et al. (2005), who reported that yogurts produced with the addition of exopolysaccharide had a softer gel structure. In addition, the hardness of yogurt produced using exopolysaccharide‐forming cultures was lower than that of yogurt produced with nonexopolysaccharide‐forming cultures, essentially owing to the incompatibility between milk proteins and exopolysaccharides (Amatayakul et al. 2006).

**TABLE 4:** Changes in the hardness, viscosity, and water holding capacity of yogurt samples during storage.

Parameters	Yogurt types	Day of storage			Mean^2^
		1	15	30	
Hardness (g)	C^1^	393.1 ± 6.3	426.6 ± 23.3	456.6 ± 13.3	425.4 ± 10.6^A^
	KG1	356.7 ± 13.6	413.9 ± 4.2	422.4 ± 1.3	397.7 ± 10.6^B^
	KG2	341.5 ± 11.8	388.8 ± 18.9	415.3 ± 8.8	381.5 ± 10.6^bc^
	KG3	340.5 ± 30.2	378.0 ± 28.5	394.7 ± 28.6	371.0 ± 10.6^C^
	Mean^2^	357.7 ± 9.2^a^	401.8 ± 9.2^b^	422.3 ± 9.2^c^	
Viscosity (mPa·s)	C^1^	1995 ± 116.7	2165 ± 208.6	2302 ± 551.4	2154 ± 157.9
	KG1	2017 ± 31.8	2040 ± 275.7	2175 ± 318.1	2077 ± 157.9
	KG2	2202 ± 286.4	1972 ± 339.3	2152 ± 222.7	2019 ± 157.9
	KG3	2005 ± 28.3	1950 ± 212.1	2139 ± 256.6	2031 ± 157.9
	Mean^2^	2055 ± 136.8	2031 ± 136.8	2191 ± 136.8	
Water holding capacity (%)	C^1^	95.00 ± 1.53	95.38 ± 0.01	95.83 ± 0.17	95.40 ± 0.37
	KG1	96.42 ± 0.01	95.99 ± 0.17	96.10 ± 0.17	96.17 ± 0.37
	KG2	96.94 ± 0.13	94.91 ± 0.59	95.43 ± 0.72	95.76 ± 0.37
	KG3	95.52 ± 0.39	94.96 ± 0.26	95.47 ± 1.15	95.32 ± 0.37
	Mean^2^	95.97 ± 0.32	95.31 ± 0.32	95.71 ± 0.32	

*Note:* Values with different letters (A–C) in the same column are significantly different (*p* ≤ 0.05). Values with different letters (a–c) in the same row are significantly different (*p* ≤ 0.05). C: control, KG1: 0.1%, KG2: 0.2%, and KG3: 0.3% kefir grain containing yogurt sample.

^1^ Mean ± standard deviation.

^2^ Mean ± standard error.

### 3.5 Gel Viscosity

Viscosity is one of the most important parameters that affects yogurt quality. During storage, the viscosity values experimental yogurt samples ranged from 1950 to 2302 mPa·s (Table 4). On the first day of storage, the control sample had the lowest value of 1995 mPa·s, and sample KG2 had the highest value of 2202 mPa·s. At the end of storage, sample C reached the highest value of 2302 mPa·s. The viscosity values of yogurt samples fortified with kefir grain powder were generally lower than those of the control sample. Despite this observation, statistical analysis revealed that the addition of kefir grain powder or storage period had no significant effect on the viscosity (*p* > 0.05). Therefore, it was concluded that the addition of kefir grain powder had a negligible effect on viscosity values of experimental yogurts. This result is consistent with the findings of Yousefvand et al. (2024) and Pham et al. (2024), who observed no significant difference in viscosity values with the addition of postbiotic powders.

### 3.6 Water Holding Capacity

The ability of the yogurt mass to retain water is one of the crucial determinants affecting quality. The separation of serum from yogurt mass is not desirable in terms of consumer appreciation. The data in Table 4 show that the WHC values of the experimental yogurt samples varied between 94.91% and 96.94%. At the beginning of storage, the lowest value was 95.0% for the control sample, whereas the highest value was 96.94% for the KG2 sample. At the end of storage (on the 30th day), the highest value was 96.10% for the KG1 sample. Only the WHC of the control sample continued to increase throughout the cold storage period. Statistical analysis showed that neither the incorporation of kefir grain powder nor the storage period had a significant effect on the WHC values (*p* > 0.05). Therefore, no significant improvement in the WHC of the experimental yogurts was achieved with the addition of kefir grain powder despite containing high amounts of exopolysaccharides. In contrast, Khider et al. (2022) observed a corresponding increase in the WHC of yogurt as the exopolysaccharide concentration increased. This discrepancy was probably a result of differences in exopolysaccharide structure. Sadighbathi et al. (2023), Pham et al. (2024) and Yousefvand et al. (2024) reported lower WHC values for yogurts fortified with postbiotic powder than our findings. This result was probably due to the high content of kefiran in kefir grain powder.

### 3.7 Viability of Yogurt Cultures

The *S. thermophilus* counts of yogurt samples ranged from log 8.50–9.84 cfu/g during storage (Table 5). At the beginning of storage, the lowest value of log 8.84 cfu/g was detected in the KG2 sample, whereas the highest value of log 9.84 cfu/g was detected in the control sample. At the end of the storage timeframe, the control and KG3 samples presented the highest values, with log 8.51 cfu/g. Statistical analysis revealed that the effects of kefir grain powder addition and storage period on *S. thermophilus* counts were insignificant (*p* > 0.05). In other words, kefir grain powder addition insignificantly altered the *S. thermophilus* count. Although the *S. thermophilus* counts decreased progressively during the storage period (except for the KG2 sample), this decrease was statistically negligible. Earlier studies carried out by Batawy and Khalil (2018), Pham et al. (2024) and Sadighbathi et al. (2023) also observed this trend, a decline in the viability of *S. thermophilus* during refrigerated storage. The numbers of *S. thermophilus* detected in the current study are consistent with earlier reports (Amatayakul et al. 2006; Akın and Akın 2016; Sadighbathi et al. 2023; Pham et al. 2024; Yousefvand et al. 2024).

**TABLE 5:** Changes in *Streptococcus thermophilus* and *Lactobacillus delbrueckii* subsp. *bulgaricus* counts of yogurt samples during storage.

Counts (log cfu/g)	Yogurt types	Day of storage		
		1	15	30
*Streptococcus thermophilus*	C^1^	9.84 ± 0.38	9.40 ± 0.23	8.51 ± 0.01
	KG1	9.19 ± 0.58	9.11 ± 0.68	8.50 ± 0.09
	KG2	8.84 ± 1.19	9.07 ± 0.74	8.50 ± 0.07
	KG3	9.32 ± 0.46	8.60 ± 0.01	8.51 ± 0.11
	Mean^2^	9.30 ± 0.26	9.05 ± 0.26	8.50 ± 0.26
*Lactobacillus delbrueckii* subsp. *bulgaricus*	C^1^	9.39 ± 0.28	8.51 ± 0.07	8.55 ± 0.08
	KG1	9.07 ± 0.74	9.07 ± 0.77	8.61 ± 0.11
	KG2	9.30 ± 0.43	9.03 ± 0.83	8.65 ± 0.09
	KG3	9.23 ± 0.55	8.56 ± 0.06	8.48 ± 0.03
	Mean^2^	9.25 ± 0.23^a^	8.79 ± 0.23^ab^	8.57 ± 0.23^b^

*Note:* Values with different letters (a, b) in the same row are significantly different (*p* ≤ 0.05). C: control, KG1: 0.1%, KG2: 0.2%, and KG3: 0.3% kefir grain containing yogurt sample.

^1^ Mean ± standard deviation.

^2^ Mean ± standard error.

During storage, the *L. delbrueckii* subsp. *bulgaricus* counts of the trial yogurts varied between log 8.48 and log 9.39 cfu/g (Table 5). At the beginning of storage, the lowest value was log 9.07 cfu/g in the KG1 yogurt, and the highest value was log 9.39 cfu/g in the regular yogurt. At the end of storage, the highest value was log 8.65 cfu/g in the KG2 sample. Statistical analysis revealed that the effect of kefir grain powder addition on *L. delbrueckii* subsp. *bulgaricus* counts was negligible (*p* > 0.05), but the effect of storage period was significant (*p* ≤ 0.05). The highest mean count during storage was determined on Day 1 (log 9.25 cfu/g), which was similar to the count determined on Day 15 (log 8.79 cfu/g) (*p* > 0.05), but significantly greater than the count determined on Day 30 (log 8.57 cfu/g). The decrease in the *L. delbrueckii* subsp. *bulgaricus* count as the storage period progressed was probably due to the notable increase in acidity. In our study, the range detected for the number of *L. delbrueckii* subsp. *bulgaricus* was greater than the range reported by Amatayakul et al. (2006) but similar to the ranges reported by Isleten and Karagul‐Yuceer (2006), Akın and Akın (2016), Sadighbathi et al. (2023), Pham et al. (2024) and Yousefvand et al. (2024).

### 3.8 Sensory Evaluation

**FIGURE 2:**
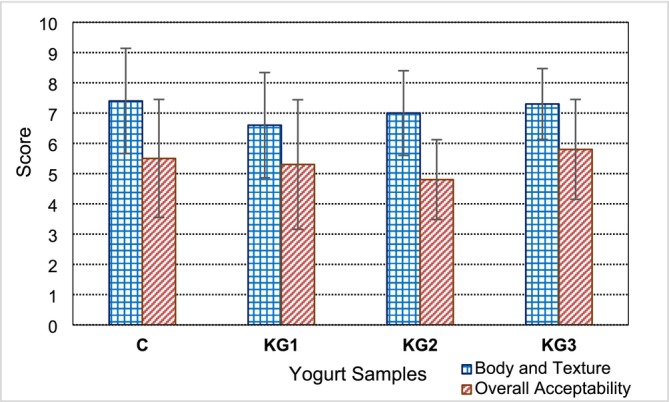
Sensory evaluation of the experimental yogurt samples (C: control, KG1: 0.1%, KG2: 0.2%, and KG3: 0.3% kefir grain containing yogurt sample).

The results of the sensory evaluation of the experimental yogurt samples carried out on the 15th day of storage are presented in Figure 2. The panelists first scored the body and textural properties of the yogurt samples and then their overall acceptability levels using a hedonic scale between 0 and 9. In the textural evaluation, the control yogurt sample received the highest score of 7.4, whereas the KG1 formulation received the lowest score of 6.6. The data obtained for the control sample were consistent with the instrumentally measured hardness and viscosity values; however, the data obtained for sample KG1 was not consistent with the instrumental results. Despite these findings, statistical analysis showed that the effects of kefir grain powder addition on the body and texture were not significant (*p* > 0.05).

In terms of overall acceptability, the panelists assigned the highest score of 5.8 to the KG3 formulation and the lowest score of 4.8 to the KG2 sample. As shown in Figure 2, the overall acceptability scores of the yogurt samples changed nonlinearly with increasing concentration of kefir grain powder. Furthermore, statistical analysis revealed that the effect of kefir grain powder addition on the overall acceptability scores of the yogurt samples was negligible (*p* > 0.05). Our observations are consistent with the results of Pham et al. (2024) and Yousefvand et al. (2024), who found that control yogurt and postbiotic formulations were equally liked by the panelists. In contrast, Sadighbathi et al. (2023) reported that postbiotic‐enriched yogurt was rated as highly acceptable by panelists.

## 4 Conclusions

In the present study, the use of dried kefir grain as an alternative postbiotic source in yogurt production was investigated. The optimum conditions for the production of kefir grains were as follows: inoculation rate: 10%, incubation temperature: 24°C, incubation time: 24 h, and shaker speed: 90 rpm. Kefir grain powder had no significant effect on the activity of starter bacteria, pH, titratable acidity, viscosity, water holding capacity, or sensory qualities of the experimental yogurts but significantly reduced the hardness values. Although the effect of kefir grain powder addition on the L* and b* values was negligible, it significantly decreased the a* values of the yogurt samples, resulting in a greener color. Unexpectedly, the viability of yogurt bacteria was not altered by the addition of kefir grain powder. Contrary to expectations, the addition of kefir grain powder did not improve either serum separation or viscosity, but it did lead to a decrease in gel hardness. However, the yogurts produced with the addition of kefir grain powder were assigned slightly higher scores by the panelists. Therefore, our study supports the hypothesis that kefir grain powder may be used as a potential postbiotic ingredient in producing yogurt that is more appealing to health‐conscious consumers without significantly altering the physicochemical properties of yogurt. This experimental yogurt, which combines the immune system‐supporting properties of both yogurt and kefir, could make a significant contribution to public health. The most important limiting factor in the study was that the maximum amount of kefir grain powder that could be used without any defects in structure was 0.3%. Therefore, studies are needed to increase the dispersibility of kefir grain powder so that it can be used in higher proportions in yogurts.

## Author Contributions

**Fatma Çoban:** data curation (equal), formal analysis (equal), methodology (equal), writing – original draft (equal). **Ezgi Demir Özer:** data curation (equal), formal analysis (equal), methodology (equal), writing – original draft (equal). **Metin Yildirim:** conceptualization (equal), methodology (equal), project administration (equal), supervision (equal), writing – original draft (equal), writing – review and editing (equal).

## Conflicts of Interest

The authors declare no conflicts of interest.

## Data Availability

The data that support the findings of this study are available on request from the corresponding author.
